# Field assessment of a model tuberculosis outbreak response plan for low-incidence areas

**DOI:** 10.1186/1471-2458-7-307

**Published:** 2007-10-26

**Authors:** Laura Freimanis Hance, Karen R Steingart, Christine G Hahn, Lisa Pascopella, Charles M Nolan

**Affiliations:** 1 Westat, Inc, Rockville, Maryland, USA; 2 Francis J. Curry National Tuberculosis Center, San Francisco, California, USA; 3 Division of Health, Idaho Department of Heath and Welfare, Boise, Idaho, USA; 4 Public Health – Seattle & King County, Seattle Washington, USA

## Abstract

**Background:**

For a regional project in four low-incidence states, we designed a customizable tuberculosis outbreak response plan. Prior to dissemination of the plan, a tuberculosis outbreak occurred, presenting an opportunity to perform a field assessment of the plan. The purpose of the assessment was to ensure that the plan included essential elements to help public health professionals recognize and respond to outbreaks.

**Methods:**

We designed a semi-structured questionnaire and interviewed all key stakeholders involved in the response. We used common themes to assess validity of and identify gaps in the plan. A subset of participants provided structured feedback on the plan.

**Results:**

We interviewed 11 public health and six community stakeholders. The assessment demonstrated that (1) almost all of the main response activities were reflected in the plan; (2) the plan added value by providing a definition of a tuberculosis outbreak and guidelines for communication and evaluation. These were areas that lacked written protocols during the actual outbreak response; and (3) basic education about tuberculosis and the interpretation and use of genotyping data were important needs. Stakeholders also suggested adding to the plan questions for evaluation and a section for specific steps to take when an outbreak is suspected.

**Conclusion:**

An interactive field assessment of a programmatic tool revealed the value of a systematic outbreak response plan with a standard definition of a tuberculosis outbreak, guidelines for communication and evaluation, and response steps. The assessment highlighted the importance of education and training for tuberculosis in low-incidence areas.

## Background

Tuberculosis (TB) is a treatable disease that causes considerable morbidity and mortality throughout the world, accounting for nearly nine million new cases and two million deaths in 2005 alone [1]. Controlling TB requires integrated public health and medical systems that serve the entire population. The United States has witnessed decreasing TB incidence since 1992, with 2006 marking the lowest number of cases (13,779 cases, 4.6 per 100,000 population) ever reported in this country [2]. Although the idea of eliminating TB in the U.S. (TB case rate < 1 per 1,000,000 population by 2010) had been discussed for decades [3,4], the recent decline in TB, after a brief resurgence in the mid-1980s to early 1990s, has given new impetus to this possibility [5,6].

Areas of low-incidence (≤ 3.5 cases per 100,000 population), where achievement of TB elimination would seem most likely, present distinct challenges to TB control including: (a) variability of local resources; (b) lack of public health and clinical TB experience; and (c) geographic barriers that may present difficulties for individuals seeking medical care [6,7]. These factors contribute to delayed recognition of infectious TB cases and higher frequency of outbreaks which have been increasingly reported [8-13]. Regional approaches may offer an innovative way to respond to these difficulties [6,7].

Four low-incidence states, Idaho, Montana, Utah, and Wyoming (2006 regional incidence rate = 1.30 cases per 100,000 population [2]), participate in a project that aims to enhance capacity among state and local health departments for controlling TB through participation in regional activities and utilization of standardized tools. Although the states differ in their TB-related epidemiology and organizational structures, they share the challenges of ensuring TB care and control in the context of decreasing TB expertise and competition for resources.

The Centers for Disease Control and Prevention (CDC) strongly encourages all states to have a written TB outbreak response plan (ORP) for the purpose of ensuring comprehensive and timely response in order to interrupt TB transmission. Many states, especially those with few TB cases, do not currently have such plans. To address the stated need for a written plan for TB outbreaks, we designed a model ORP based on existing plans for TB and other communicable diseases and key informant interviews with local, state, and national TB experts. Representatives from the four low-incidence states and the Division of Tuberculosis Elimination (DTBE), CDC also provided input. The ORP is designed as a template permitting customization by a specific jurisdiction.

Prior to dissemination of the ORP, we became aware of a TB outbreak underway in Idaho. The outbreak presented an opportunity to perform a field assessment to validate and refine the tool. During October 2005–February 2006, four cases of TB disease were reported in Boise. Three of the patients were homeless and all four patients had matching *Mycobacterium tuberculosis *genotypes by spacer oligonucleotide (spoligotyping), mycobacterial interspersed repetitive unit analysis, and restriction fragment length polymorphism analysis. The (local) district TB program led the outbreak response, while the state TB program and CDC provided technical assistance and laboratory services. Other states in the area were alerted and genotyping information was provided. A TB-specific plan was not used to guide response activities. In April 2006, three investigators visited the area to conduct an assessment of the ORP. The purpose of the assessment was to ensure that the plan included essential elements to help TB professionals recognize and respond to outbreaks. We were also interested in learning what education and training needs arose during the outbreak.

## Methods

We followed recommended methods for conducting key informant interviews [14]. Key informants were individuals who knew of or were involved in the outbreak, as identified by the state TB controller and district management staff. The primary intent of the assessment was to enhance a current, on-going public health activity (TB control), by improving a specific tool to help public health staff respond to TB outbreaks. Study participants were public health professionals and community health and social service providers; patient data were not used. Nonetheless, we followed procedure to ensure that participation was voluntary, that the purpose of the assessment was understood by participants, and that responses remained confidential. Toward that end, when requesting appointments and again at the time of the interview, we informed respondents that participation was voluntary and explained the purpose and duration of the interview, the benefits that could be expected from participation (i.e., an improved ORP), and the confidential nature of the interview.

We created and pilot-tested a questionnaire to guide the interviews. In addition, we requested review of the questionnaire from a state epidemiologist who worked in a related area of public health. Based upon experience gained in the pilot and specific feedback on the instrument, the questionnaire was finalized. The interview questions were designed to discuss TB control, leadership, and organizational issues previously identified as essential elements in the ORP, as well as identify gaps. In particular, we focused on the following elements: identification of the outbreak, legal authority, notification, roles and responsibilities, resources, communication, community partnerships, and evaluation. We tailored questions as appropriate for the different groups of interviewees (i.e., public health professionals and community health and social service providers), see Additional file 1.

We conducted semi-structured interviews in-person and by phone when face-to-face meetings were not feasible due to distance or time. Four state public health laboratory professionals provided a consensus response. Two respondents, one state and one national health official, had seen earlier drafts of the ORP. All other interviews were conducted without any prior knowledge of the plan. In addition, we asked interviewees from the district, state, and CDC TB programs to review the ORP and provide structured feedback on specific sections (definition of a TB outbreak, notification, local and state public health responsibilities, communication, and evaluation). As we estimated it would take several hours to appraise the ORP and reply to the written questionnaire, we asked only a subset of interviewees to participate in this phase of the assessment.

### Data extraction and analysis

We used an inductive process to identify and compare concepts and insights from patterns in the data [15]. First, two trained interviewers analyzed the interview transcripts and independently color-coded responses to the questions allowing the identification of themes. We then compared the color-coding and independently derived themes to generate a single list of themes. Differences in opinion were resolved by consensus. Finally, we discussed the themes with the state TB controller for validation, comment, and clarification. What emerged was a final list of central themes that was used as a basis for determining whether these themes were represented and adequately addressed in sections in the ORP.

## Results

We interviewed all 17 stakeholders identified as being involved with the response. Specifically, we interviewed 11 (65%) public health professionals (four state, six district, and one national representative) and six (35%) community members (two physicians, two infection control practitioners, and two homeless shelter employees), as summarized in Table 1. We conducted all but two of the interviews in-person.

**Table 1:** Key informants and their tuberculosis-related responsibilities

**Jurisdiction/area**	**Type of interview**	**Responsibilities**
State	In-person	Provides consultation in clinical and public health matters
	In-person	Under unusual circumstances, serves as lead epidemiologist and liaison with the local health districts and the state public health laboratory. (Provided public health consultation during the TB outbreak while the TB controller was on leave of absence)
	In-person	Provides oversight for surveillance and control of communicable diseases, including TB
	In-person*	Provides TB-related laboratory services; is responsible for processing TB samples
District	In-person	Performs TB case and contact investigations; reports TB cases; and advises about isolation
	In-person	Provides TB-program oversight and supervises nurse TB case manager
	In-person	Issues media releases and manages website with TB-specific materials prepared by the epidemiologists
	In-person	In this situation, provided interim management of epidemiologic activities
	In-person	Manages TB cases and provides treatment for latent TB infection
	In-person	Performs TB case and contact investigations and reports TB cases
CDC	Phone	Provides technical assistance in TB control and prevention to nine states in the Pacific Northwest and Rocky Mountains. CDC provides state funding for TB prevention and control activities
Community	In-person	Provides patient care and consultation
	In-person	Provides patient care and consultation
	Phone	Coordinates hospital infection control
	In-person	Coordinates hospital infection control
	In-person	Facilitates TB education
	In-person	Facilitates TB education; participates in TB clearance program

* Four laboratory professionals provided a consensus response. CDC, Centers for Disease Control and Prevention; TB, tuberculosis.

Overall, the central themes from the outbreak response that emerged during the key informant interviews were reflected in sections of the original ORP (Table 2). Four of the central themes (noted in italics below) are described in more detail in order to illustrate characteristics of the ORP. In particular, we describe the outbreak response through participants' own accounts (the outbreak) and then compare these accounts with guidance provided in the original ORP (the ORP).

**Table 2:** Selected themes identified during key informant interviews

**Themes**	**Related section(s) in the outbreak response plan**	**Observations**
Identification of the outbreak relied on both epidemiologic methods and genotyping information.	Definition of a TB outbreak; ten steps to take when an outbreak is suspected; exceptional TB circumstances; data management; glossary	The district became aware of the outbreak by epidemiologic methods; the state, by genotyping matches. A standard TB outbreak definition was not used.
Legal authority for responding to the outbreak was clearly established.	Legal authority; indications for initiating the plan; de-activation of the TB outbreak response plan	All respondents agreed that legal authority for the outbreak rested with the district; a TB-specific plan was not used to guide the response.
Technical assistance was requested when the outbreak was first identified.	Notification and request for assistance	The state TB program notified the Division of TB Elimination, CDC, allowing technical assistance to be deployed in a timely manner.
Additional resources were needed to respond to the outbreak.	Composition of the outbreak response team; public health roles and responsibilities; sources of additional staffing; training and education	In low-incidence areas, multiple roles are often filled by one individual; additional employees from other public health programs were brought in to help with the outbreak, but lacked TB training.
Communication relied on multiple channels, both formal and informal.	Guidelines for internal and external communication; risk communication checklist	The state TB controller and district epidemiologists communicated using standard operating procedures with health professionals; however, within the public health sector, staff relied on informal mechanisms for communication across multiple jurisdictions.
Contributions by community members were an integral part of the outbreak response.	Community partnerships	All community members had knowledge of and were engaged in response activities (e.g., care of TB patients, education of co-workers, TB screening at the shelter, conduction of contact investigations) at their respective facilities.
The basics of TB and interpretation of genotyping information were important areas for education.	Training and education	The assessment highlighted the importance of continued TB education and training in low-incidence areas.

CDC, Centers for Disease Control and Prevention; TB, tuberculosis.

Of the seven public health interviewees who provided structured feedback on the ORP, almost all agreed that the ORP would have been useful to have had during the outbreak. The feedback included specific suggestions for improving the ORP. These ideas, as well as comments from the key informant interviews, helped us make the final ORP more usable and relevant and are described below.

### Central themes

#### Identification of the outbreak relied on both epidemiologic methods and genotyping information

##### The outbreak

Most respondents were aware of the outbreak and described in "real time" how the outbreak was recognized. Four of the six district respondents identified the outbreak through traditional epidemiologic methods and two respondents were uncertain how the outbreak was first recognized. The district began taking local action when staff identified homelessness as a common risk factor among the TB patients. The CDC respondent and all four state respondents became aware of the outbreak as a result of reviewing genotyping information that indicated matching TB strains. This in turn led state epidemiologists to investigate the situation further by reviewing epidemiologic data with district staff. The state and CDC subsequently alerted other states within the region. A standard TB outbreak definition had not been used and there appeared to be disagreement among some respondents about whether and when an outbreak had actually occurred. Most respondents felt that a standard TB outbreak definition might help with early recognition of future TB outbreaks and coordination of response activities.

##### The ORP

**Figure 1:**
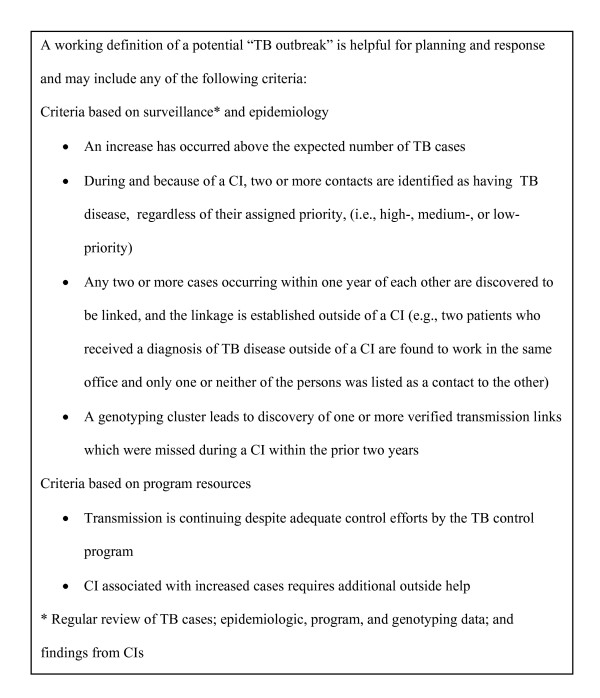
**Definition of a tuberculosis outbreak**. CDC, Centers for Disease Control and Prevention; CI, Contact investigation; TB, tuberculosis. (Figure1_Defintion_TB_Outbreak; pdf; Definition of a tuberculosis outbreak; box with definition).

Understanding that definitions of a TB outbreak are relative to the local context, the ORP proposes a TB outbreak definition which incorporates both epidemiologic methods and genotyping information, Figure 1. The ORP explains that outbreak cases can be distinguished from other cases only when certain associations in time, location, patient characteristics, or *Mycobacterium tuberculosis *attributes (e.g., drug resistance or genotype) become apparent. The ORP recommends that in low-incidence jurisdictions, any temporal cluster of cases should be considered suspicious for an outbreak.

#### Communication relied on multiple channels, both formal and informal

##### The outbreak

We were interested in determining who served as the lead spokesperson with different audiences (e.g., community health providers, the public, and TB professionals from other states in the region) and whether written guidelines were used. As per standard operating procedures, the district and state public information officers managed communication efforts for the public, and the district epidemiologists and state TB controller directed communication activities involving health providers. The laboratory group uses an alert system to convey urgent information about communicable diseases, including TB, to other laboratories; however, the system was not deployed in this situation. The state TB controller alerted public health personnel from jurisdictions within the state and both the state TB controller and CDC representative communicated with TB program staff in other states. According to the respondents, communication with the public relied on written guidelines; however, communication within the governmental public health system used an informal process.

##### The ORP

The importance of identifying and preparing key spokespersons and coordinating information activities via a written communication plan is emphasized. This is especially important when multiple jurisdictions within a state and/or multiple states are involved. The ORP provides a TB outbreak communication checklist and worksheet for developing key public health messages, see Additional file 2.

#### Contributions by community members were an integral part of the outbreak response

##### The outbreak

All six community members were involved with various aspects of the response. The physicians and infection control practitioners reported TB cases to the public health district, cared for TB patients, advised about infection control, and educated co-workers about TB. One respondent was involved in a contact investigation of patients and employees exposed to an infectious TB patient at her institution. One respondent wrote an article about TB for a staff electronic newsletter that was widely distributed. The two homeless shelter employees participated by educating clients and conducting a TB screening program.

##### The ORP

Partnerships with hospitals, private providers, and community groups, such as homeless advocates, are essential to successfully reach persons with TB disease and infection. For example, health department staff may lose patients because of social, cultural, and linguistic barriers. The forging of partnerships can help overcome these problems. The ORP reinforces the importance of sustaining partnerships and provides a partial list of suggested partner organizations.

#### The basics of tuberculosis and interpretation of genotyping information were important areas for education which emerged during the outbreak

##### The outbreak

Several community respondents expressed concern about the risk of transmission of *Mycobacterium tuberculosis *from transient individuals (e.g., migrant farm-workers and homeless individuals), pointing to the need for further education about TB transmission, the difference between infection and disease, and cultural competency. Public health respondents identified the use and interpretation of genotyping data and basic TB education for non-TB staff as their top training needs. (This information was conveyed to the Francis J. Curry National TB Center, one of four CDC-funded U.S. Regional Training and Medical Consultation Centers, for consideration in preparing future TB curricula. "Genotyping, practical applications and interpretation" is now an educational offering).

##### The ORP

Education of TB program and other public heath staff, community providers, and laboratory professionals should be ongoing, but often needs to be specially arranged during a TB outbreak. Resources are available through CDC and the Regional Training and Medical Consultation Centers.

### Improvements to the ORP

Several respondents recommended including more information in the section on evaluation and one respondent suggested adding advice about what to do if a TB outbreak is suspected. Based on these comments and additional suggestions received during the field assessment, we revised the ORP as follows: expanded the section on evaluation and added a checklist with questions, see Additional file 3; added "ten steps to take when a TB outbreak is suspected", see Additional file 4; explained the reasons why notification of TB outbreaks to CDC is important (for the purposes of obtaining assistance and documenting outbreaks as the U. S. moves toward TB elimination); simplified the section on roles and responsibilities of participants in the outbreak response; emphasized the important role of the public health laboratory in outbreak response; added information on inter-jurisdictional communication and data sharing; expanded the glossary; and included a table of contents. The final version of the ORP is described below.

### The final outbreak response plan

Although, all real-time outbreak response activities were reflected in the initial ORP, we incorporated many of the suggestions for improvement into the final version of the plan. The goals of the ORP are (a) to identify all outbreak-associated cases; (b) to initiate contact investigations in a timely manner; and (c) to identify infected persons for evaluation of TB and ensure appropriate follow-up. The ORP outlines in a concise manner the following elements considered essential for recognition of and response to TB outbreaks: definition of a TB outbreak; indications for initiating the plan; legal authority; composition of the response team; procedures for notification and request for assistance; local and state public health responsibilities; data management; communication; training and education; community partnerships; and evaluation [16]. In addition, the ORP includes checklists for risk communication and evaluation, a glossary of outbreak-related terms, and sections on "ten steps to take when a TB outbreak is suspected" and "exceptional TB circumstances," situations of sufficient concern to prompt activation of the ORP. The "model tuberculosis ORP for low-incidence areas: a customizable template [17]" and a customizable "TB program manual template [18]" that presents national TB control guidelines, including guidance on contact investigation, for use in the field are available from the Francis J. Curry National Tuberculosis Center.

## Discussion

Our assessment of a TB outbreak response plan for low-incidence areas demonstrates that (1) almost all the main response activities were reflected in the plan; (2) the plan added value by providing a standard TB outbreak definition and guidelines for response activities, in particular, communication and evaluation, subjects for which a written protocol was lacking during the actual response; and (3) basic education about TB and the interpretation and use of genotyping data were important needs. Stakeholders suggested adding a section to the plan with specific steps to take when an outbreak is suspected and an evaluation checklist. Idaho public health staff indicated that having a written ORP would be useful for future outbreaks. A written ORP provides a systematic approach for identification of outbreaks and coordination of the response in any area, but it becomes critical in low-incidence areas, where pubic health workers are usually "generalists" who work with several different programs (e.g., immunization and Women, Infants, and Children [WIC]) and may be less familiar with TB.

Our assessment had several strengths. First, we identified and interviewed all major participants in this outbreak, thus minimizing selection bias, an inherent shortcoming of key informant interviews [14]. Second, we used trained interviewers, who after establishing rapport, used careful phrasing and probing techniques to encourage interviewees to explain the reasons for their statements. Third, two interviewers independently identified and then compared central themes. Finally, we validated central themes with the state TB controller.

Our assessment also had limitations. A primary shortcoming was the possibility of interviewer bias. Although, we attempted to minimize bias by the use of trained interviewers, nonetheless, we recognize this as a limitation of key informant interviews [14]. Second, our findings are based on the perceptions of participants, rather than on objective methods. Third, by requesting feedback on the ORP from a subset of public health professionals, rather than the entire group, we may have missed ideas for improving the ORP. And finally, we recognize the results from this Idaho region may not be generalizable to other areas. However, despite these limitations, we believe the assessment led to improvements in the ORP as a customizable tool for use by TB programs in other low-incidence areas.

TB outbreaks pose immediate threats to the health of communities and, over time, increase the number of individuals with latent TB infection who may later develop TB disease. Often, there is a delay in recognizing outbreaks and in responding effectively when they occur [16]. This assessment identified the need for a standard TB outbreak definition. Information from traditional epidemiologic methods and newer genotyping data were both considered to be essential elements of the definition. In the Idaho outbreak, district staff relied on epidemiologic methods to trigger action, whereas state staff considered genotyping matches to provide the clue that led to suspicion of an outbreak. The use of genotyping data has been shown to detect TB transmission events and help guide public health interventions [19-21]. We propose a broad definition of a TB outbreak, to provide guidance and discretion in deciding when to declare an "outbreak". In a low-incidence area, we recommend setting a low threshold for triggering the response and then deciding for each instance whether a stronger response is warranted.

Ideally, we would have liked to conduct a formal evaluation of the ORP by utilizing a post-intervention study design. In this scheme, we would plan an intervention by distributing the ORP across the region, holding training sessions with local and state public health staff, and then measuring how response activities changed – quantitatively and qualitatively – after the intervention. Although there have been increased reports of outbreaks in low-incidence areas, TB outbreaks are still a relatively rare event. This type of evaluation will have to await the appropriate condition (TB outbreak) and when it occurs, should be performed to continue to improve TB control and work towards TB elimination.

## Conclusion

In low-incidence areas, an ORP may be a useful management tool that provides a systematic mechanism for recognition and response to TB outbreaks, planning for additional resources, and prioritizing training. Feedback from end users provided useful information for refining this kind of tool. The assessment highlighted the importance of continued TB education and training in low-incidence areas.

## Competing interests

The author(s) declare that they have no competing interests.

## Authors' contributions

LFH and KRS conceived of and designed the study, conducted key informant interviews, analyzed and interpreted the data, and helped draft and revise the manuscript. CGH interpreted the data and helped draft the manuscript. LP conceived of the study, interpreted the data, and helped draft and revise the manuscript. CMN conducted key informant interviews and helped draft the manuscript. All authors read and approved the final manuscript.

## Pre-publication history

The pre-publication history for this paper can be accessed here:



## Supplementary Material

Additional file 1 **Outbreak response plan assessment questionnaire**. key informant questions; list of questions) Click here for file

Additional file 2 **Risk communication checklist**. Risk communication checklist; table of questions for risk communication planning) Click here for file

Additional file 3 **Evaluation checklist**. evaluation questions) Click here for file

Additional file 4 **Ten steps to take when a tuberculosis outbreak is suspected**. Ten steps to take when a tuberculosis outbreak is suspected; steps to take when a TB outbreak is suspected) Click here for file

## References

[B1] World Health Organization (2007). Global tuberculosis control: surveillance, planning, financing. WHO report 2007. WHO/HTM/TB/2007.376.

[B2] Centers for Disease Control and Prevention Reported tuberculosis in the United States, 2006. http://www.cdc.gov/tb/surv/surv2006/pdf/FullReport.pdf.

[B3] Centers for Disease Control (CDC) (1989). A strategic plan for the elimination of tuberculosis in the United States. MMWR Morb Mortal Wkly Rep.

[B4] Frost W (1937). How much control of tuberculosis?. Am J Pub Health.

[B5] (1999). Tuberculosis elimination revisited: obstacles, opportunities, and a renewed commitment. Advisory Council for the Elimination of Tuberculosis (ACET). MMWR Recomm Rep.

[B6] Geiter L, Institute of Medicine, Committee on the Elimination of Tuberculosis in the United States (2000). Tuberculosis elimination and the changing role of tuberculosis control programs. Ending neglect: the elimination of tuberculosis in the United States.

[B7] Centers for Disease Control (CDC) (2002). Progressing towards tuberculosis elimination in low-incidence areas of the United States. MMWR.

[B8] Centers for Disease Control and Prevention (2000). Drug-susceptible tuberculosis outbreak in a state correctional facility housing HIV-infected inmates---South Carolina, 1999--2000. MMWR.

[B9] Centers for Disease Control and Prevention (2001). Cluster of tuberculosis cases among exotic dancers and their close contacts---Kansas, 1994--2000. MMWR.

[B10] McLaughlin SI, Spradling P, Drociuk D, Ridzon R, Pozsik CJ, Onorato I (2003). Extensive transmission of Mycobacterium tuberculosis among congregated, HIV-infected prison inmates in South Carolina, United States. Int J Tuberc Lung Dis.

[B11] Centers for Disease Control and Prevention (2003). Public health dispatch: tuberculosis outbreak in a homeless population--Portland, Maine, 2002-2003. MMWR.

[B12] Centers for Disease Control and Prevention (2004). Tuberculosis outbreak in a low-incidence state--Indiana, 2001-2004. MMWR.

[B13] Phillips L, Carlile J, Smith D (2004). Epidemiology of a tuberculosis outbreak in a rural Missouri high school. Pediatrics.

[B14] United States Agency for International Development Center for Development and Information Evaluation (1996). Conducting key informant interviews. http://www.usaid.gov/pubs/usaid_eval/pdf_docs/pnabs541.pdf.

[B15] Glaser BG (1965). The constant comparative method of qualitative analysis. Social problems.

[B16] Taylor Z, Nolan CM, Blumberg HM, American Thoracic Society, Centers for Disease Control and Prevention, Infectious Diseases Society of America (2005). Controlling tuberculosis in the United States. Recommendations from the American Thoracic Society, CDC, and the Infectious Diseases Society of America.

[B17] Model tuberculosis outbreak response plan for low-incidence areas: a customizable template. http://www.nationaltbcenter.edu/resources/tb_orp_lia.cfm.

[B18] The tuberculosis program manual template. http://www.nationaltbcenter.edu/resources/tb_manual_template.cfm.

[B19] Curtis AB, Ridzon R, Novick LF, Driscoll J, Blair D, Oxtoby M, McGarry M, Hiscox B, Faulkner C, Taber H, Valway S, Onorato IM (2000). Analysis of Mycobacterium tuberculosis transmission patterns in a homeless shelter outbreak. Int J Tuberc Lung Dis.

[B20] McElroy PD, Sterling TR, Driver CR, Kreiswirth B, Woodley CL, Cronin WA, Hardge DX, Shilkret KL, Ridzon R (2002). Use of DNA fingerprinting to investigate a multiyear, multistate tuberculosis outbreak. Emerg Infect Dis.

[B21] Small PM, Hopewell PC, Singh SP, Paz A, Parsonnet J, Ruston DC, Schecter GF, Daley CL, Schoolnik GK (1994). The epidemiology of tuberculosis in San Francisco. A population-based study using conventional and molecular methods. N Engl J Med.

