# Supplementary material for: Field assessment of a model tuberculosis outbreak response plan for low-incidence areas
Source: BMC Public Health. 2007 Oct 26;7:307. doi: 10.1186/1471-2458-7-307 (PMC2194699; doi:10.1186/1471-2458-7-307)
Supplement: Additional file 1 — Outbreak response plan assessment questionnaire. key informant questions; list of questions) [file 1471-2458-7-307-S1.pdf]

Outbreak response plan assessment questionnaire

1. I would like to begin by asking you what are your TB-specific functions?
2. What is your role in the response to the current cluster of TB cases around Boise?
3. Do you know how the cluster of TB cases was identified?
3A.Do you know if genotyping data were used in identifying the cluster of TB cases?
4. Are you aware of specific criteria for identifying a TB case cluster or TB outbreak?
5. How did you personally become aware of this cluster of TB cases?
6. Do you know if the State Health Department was notified?
6A. Do you know how long it took to notify the State Health Department?
6B. Do you know how this was notification done?
7. Do you know if CDC was notified?
7A. Do you know how long it took to notify CDC?
7B.Do you know how this notification was done?
7C. Do you know if there was a "Report of Tuberculosis Transmission (RTT)" form sent to CDC?
8. Are you using a written plan to guide the response to the cluster of TB cases, for example, a plan that addresses case management, data management, contact investigation, and education and training? If so, could we have a copy of the plan? (Note to readers: this question refers to a plan that may currently exist, rather than the Outbreak Response Plan described in this paper).
9. Has the plan (mentioned above) been helpful?
10. Are there areas in the response to the cluster of TB cases that the plan (mentioned above) does not address and you wish the plan did address?
11. Was there a team identified for the response to the cluster of TB cases?
11A.Was it a state, local (district), or combined team?
11B. Were you a member of the team?
12. We want to know in which activities you participated and if you were the lead person responsible for any of these specific activities:
A. TB case management
B. Contact investigation and follow-up
C. Ensuring treatment for latent TB infection
D. Education to other public health staff and the community
F. Liaison with community providers
G. Reviewing charts for sputum smear status, symptoms, chest x-ray, and date of diagnosis
H. Advising about infection control
I. Data management, such as review and analysis of epidemiologic and genotyping data
12A.Please let me know of other activities which you were involved in, but I did not ask about.
13. Do you know if additional resources were brought in to help with the response to the TB case cluster?
14. What did you see as the three most important staffing challenges in responding to the cluster of TB cases?
15. Do you know if the laboratories in the area were notified that there was a cluster of TB cases in the community?

15A.Do you know if laboratories were able to meet the turn around times needed by the TB program and community health providers?
16. During the response to the cluster of TB cases in the community, do you know if there were guidelines, written or verbal, about who was responsible for internal communications, within the District or within the State Health Department?
17. During the response to the cluster of TB cases in the community, do you know if there were guidelines, written or verbal, about who was responsible for external communications, such as with the media, the public, community healthcare providers etc?
18. What education and training issues emerged during the response to the cluster of TB cases?
19. Do you know if any legal issues emerged during the response to the cluster of TB cases?
20. Are you aware of any TB cases related to the current TB cluster that have been identified in other public health jurisdictions, within Idaho or out-of-state?
20A. How did you become aware of the TB cases in other jurisdictions?
20B. The next question concerns communication WITHIN Idaho. After you became aware of TB cases in other public health jurisdictions, please describe how you communicated with these jurisdictions, including local and state, about potentially linked TB cases?
20C. The next question concerns communication OUTSIDE Idaho. After you became aware of TB cases in other public health jurisdictions, please describe how you communicated with these jurisdictions, including local and state, about potentially linked TB cases?
20D. Do you have a written agreement you complete before communicating with other jurisdictions about TB cases?
21. Do you have any additional comments?

CDC, Centers for Disease Control and Prevention; TB, tuberculosis
