# Supplementary material for: Field assessment of a model tuberculosis outbreak response plan for low-incidence areas
Source: BMC Public Health. 2007 Oct 26;7:307. doi: 10.1186/1471-2458-7-307 (PMC2194699; doi:10.1186/1471-2458-7-307)
Supplement: Additional file 2 — Risk communication checklist. Risk communication checklist; table of questions for risk communication planning) [file 1471-2458-7-307-S2.pdf]

Risk communication checklist

1. Planning

	Yes	No	No need
Does the TB program have an outbreak communication plan for public information and media, partner, and stakeholder relations?			
If yes, does the plan have the following elements:			
Designated line and staff responsibilities for the public information team?			
Information verification and clearance/approval procedures?			
Agreements on information release authorities (who release what/when/how)?			
Regional and local media contact list (including after-hours news desks)?			
Designated spokespersons for public health issues in an outbreak?			
Identified vehicles of information dissemination to public, stakeholders, and partners (e.g., email list serves, broadcast fax, press releases)?			
Have you coordinated your planning with other organizations?			
Have designated spokespersons received media training and risk communication training?			

2. Messages and audiences

	Yes	No	No need
Have you identified special populations (e.g., elderly, first language other than English, Tribal communities, border populations)? List any specific sub-populations that need to be targeted with specific messages during a public health emergency related to TB (e.g., Tribal nations, persons with chronic respiratory illness, seniors).			
Have you developed topic-specific, pre-crisis TB materials for outbreaks, or identified sources of these materials (if needed): Topic fact sheet (e.g., description of an outbreak investigation, transmission of TB, treatment, etc.)?			
Resource fact sheets and web links for media/public/partners to obtain additional information?			
Recommendations for affected populations?			
List of subject matter experts outside your organization, to speak to public/media regarding your activities during an outbreak?			

3. Messenger

	Yes	No	To do
Have you identified TB partnership spokespersons for media and public appearances during an outbreak?			
If yes, have you:<br>Ensured that spokespersons understand their roles and responsibilities and will incorporate them into their expected duties during the outbreak?			

(Selected elements of TB risk communication. For further information about risk communication, visit CDcynergy at http://www.cdc.gov/communication/cdcynergy.htm.)

TB, tuberculosis

Single Overriding Communications Objective (SOCO)

In one BRIEF paragraph, state the key point or objective you want to accomplish by doing the interview. This statement should reflect what you, the author or speaker, would like to see as the lead paragraph in a newspaper story or broadcast report about your topic.

What are the three or four facts or statistics you would like the public to remember as a result of reading or hearing about this story?

Who is the main audience or population segment you would like this message to reach?
Primary: Secondary:

What is the ONE message you want the audience to take away from this interview/report?

Who in your office will serve as the primary point of contact for the media?
Name: Phone: Email: Date(s) and time(s) available: Date: Time:
