# Supplementary material for: Field assessment of a model tuberculosis outbreak response plan for low-incidence areas
Source: BMC Public Health. 2007 Oct 26;7:307. doi: 10.1186/1471-2458-7-307 (PMC2194699; doi:10.1186/1471-2458-7-307)
Supplement: Additional file 3 — Evaluation checklist. evaluation questions) [file 1471-2458-7-307-S3.pdf]

Evaluation checklist

1. Was the outbreak identified in a timely manner so transmission was interrupted?

2. Was a Tuberculosis Outbreak Response Team (TORT) assembled?

3. Did the TORT composition include all appropriate representatives from the public health sector, as well as other stakeholders? If not, who was missing and why?

4. Did the TORT enhance the process?

5. Were roles clearly identified for all TORT members?

6. Did all TORT members have the information needed for them to perform their responsibilities?

7. Were internal communication mechanisms established? Did they work? If not, why?

8. Were external communication mechanisms established? Did they work? If not, why?

9. Are the findings and recommendations resulting from the outbreak response being implemented? If so, describe. If not, what are the reasons for not implementing these recommendations?
