# Supplementary material for: Field assessment of a model tuberculosis outbreak response plan for low-incidence areas
Source: BMC Public Health. 2007 Oct 26;7:307. doi: 10.1186/1471-2458-7-307 (PMC2194699; doi:10.1186/1471-2458-7-307)
Supplement: Additional file 4 — Ten steps to take when a tuberculosis outbreak is suspected. Ten steps to take when a tuberculosis outbreak is suspected; steps to take when a TB outbreak is suspected) [file 1471-2458-7-307-S4.pdf]

Ten steps to take when a tuberculosis outbreak is suspected

1. Convene the potential outbreak team with representation from local, regional (if applicable), state, and public health laboratory. Identify potential consultants, including CDC. Review legal issues, authority, and roles, and identify the lead agency.

2. Review epidemiologic and genotyping data and establish what is needed to answer the question, "Has a TB outbreak occurred?" Refer to criteria in “Definition for TB outbreak”.*

3. Clarify mechanisms for internal communication among the outbreak team (remember the team likely includes representatives from several agencies and institutions); in particular, describe channels of communication regarding new information about suspected cases, contact investigations, and laboratory data, including genotyping results.

4. Identify media spokesperson(s).

5. Review guidelines for contact investigations and ensure that protocols exist for other potential outbreak response activities.

6. Identify additional resources that may be needed, including financial resources and staffing. Discuss potential sources to obtain additional resources.

7. Enhance surveillance for TB cases (remember that this will often be associated with notification of health care providers, as noted below).

8. Decide whether or not to issue a media release, health alerts to providers, or make special contact with certain groups depending on the initial epidemiology (e.g., corrections, homeless shelters, parents at a school, etc).

9. Provide basic TB education to public health staff and plan for health care provider training and education.

10. Decide when to contact community partners.

*We recommend the decision to declare a TB outbreak and initiate the outbreak response plan be made by the state TB controller/manager or local TB controller, depending on authority for TB control in the jurisdiction. The decision should be made after consultation with local and state epidemiologists and other TB program staff. The information gathered in the “ten steps” may assist with making the decision.

CDC, Centers for Disease Control and Prevention; TB, tuberculosis.
